# *AHR* promoter variant modulates its transcription and downstream effectors by allele-specific *AHR*-SP1 interaction functioning as a genetic marker for vitiligo

**DOI:** 10.1038/srep13542

**Published:** 2015-09-15

**Authors:** Xiaowen Wang, Kai Li, Ling Liu, Qiong Shi, Pu Song, Zhe Jian, Sen Guo, Gang Wang, Chunying Li, Tianwen Gao

**Affiliations:** 1 Department of Dermatology, Xijing Hospital, Fourth Military Medical University, Xi’an, Shaanxi, China

## Abstract

Vitiligo is an acquired depigmentation disorder largely caused by defective melanocyte- or autoimmunity-induced melanocyte destruction. The aryl hydrocarbon receptor (AHR) is essential for melanocyte homeostasis and immune process, and abnormal AHR was observed in vitiligo. We previously identified the T allele of *AHR* −129C > T variant as a protective factor against vitiligo. However, biological characterization underlying such effects is not fully certain, further validation by mechanistic research is warranted and was conducted in the present study. We showed that −129T allele promoted *AHR* transcriptional activity through facilitating its interaction with SP1 transcription factor (SP1) compared with −129C allele. We subsequently found reduced peripheral *AHR* and *SP1* transcript expressions in vitiligo and a negative correlation of *AHR* level with disease duration. We also investigated AHR-related cytokines and observed increased serum TNF-α concentration and diminished serum levels of IL-10 and TGF-β1 in vitiligo. Further genetic analysis showed that -129T carriers possessed higher levels of *AHR* and IL-10 than −129C carriers. Therefore, our study indicates that the modulation of *AHR* transcription by a promoter variant has a profound influence on vitiligo, not only advancing our understanding on AHR function but also providing novel insight into the pathogenesis of degenerative or autoimmune diseases including vitiligo.

Vitiligo is a chronic depigmentation disorder resulting from melanocyte destruction. The incidence of vitiligo is approximately 0.5 ∼ 8% worldwide, and over 50% of the patients develop the disease between the ages of 10 and 30 years^1^. Vitiligo deeply affects both the physical and mental health of patients, the course and treatment response of which are highly variable^2^. Contributing factors for the initiation of vitiligo are unknown, although genetic susceptibility, autoimmunity, oxidative stress and melanocyte-intrinsic abnormalities have been implicated^1^. Accumulating data emphasize the crucial role of melanocyte-inherent defects in vitiligo, with evidence of aberrant melanogenesis pathway and impaired melanocyte development^1^,^3^. Previous studies have showed that abnormality of the rate-limiting enzymes in melanin synthesis process, including tyrosinase (TYR) and tyrosinase-related protein (TYRP), may induce excessive toxic metabolites and trigger cellular damage in vitiligo^2^. In addition, defection in stem cell factor/stem cell factor receptor (SCF/C-Kit) melanocyte survival pathway has been suggested to contribute to melanocyte apoptosis in vitiligo^2^,^4^,^5^. Besides directly inducing apoptosis, melanocyte-inherent aberrations could further initiate or amplify the autoimmune damage in vitiligo^6^,^7^,^8^,^9^.

The aryl hydrocarbon receptor (AHR) is a ligand-activated transcription factor and belongs to the basic-helix-loop-helix family^10^. Upon binding ligand, AHR translocates into the nucleus to govern target genes^11^. AHR is well characterized for immune regulation through mediating T-cell differentiation and cytokine milieu^12^,^13^, and more recently, scientific evidence strongly supports that AHR is vital to melanocyte homeostasis. Activation of AHR pathway stimulated melanogenesis by improving expressions of TYR and TYRP in human melanocytes^14^. *Ahr*-deficient mice showed decreased melanocyte number and hypopigmentation in the tail skin caused by reduced levels of SCF and C-Kit^15^. Additionally, significantly declined expressions of AHR and its target genes were observed in the epidermis of vitiligo patients^16^. These data imply that mutation or dysfunction of AHR might be involved in vitiligo.

In consideration of the critical role of AHR in both melanocyte and cellular immunity and aberrant AHR pathway in vitiligo, we previously evaluated the potential association between *AHR* polymorphisms and vitiligo susceptibility. Our data demonstrated that the T allele of −129C > T variant (rs10249788) in the *AHR* promoter region is associated with a protective effect on vitiligo in Han Chinese populations^17^, which might be a functional variation through altering *AHR* transcription process. The promoter of human *AHR* gene lacks TATA and CCAAT boxes but possesses several putative SP1 transcription factor (SP1) binding sites within a highly GC-rich region^18^. SP1 is a Cys_2_/His_2_-type zinc-finger transcription factor that binds to GC box elements (5′-GGGCGG-3′) within promoter region^19^. SP1 is particularly important to the TATA-less genes, which regulates transcription of multiple target genes involved in cell growth, differentiation, apoptosis and immune response^20^. Early researches have revealed that SP1 dominates the maximal constitutive activity and basal expression of *AHR* gene *via* binding to these GC-rich motifs^21^. The abnormal interaction between SP1 and *AHR* promoter is responsible for *AHR* down-regulation in human diseases^22^,^23^. As the −129C > T polymorphism is in the core promoter region of *AHR* gene and is juxtaposed 5′ to the SP1 recognition sequence, we therefore hypothesized that the *AHR* −129C > T polymorphism could influence its transcription and downstream effectors in melanocyte biology or immune system, which could further affect the development of vitiligo. In the present study, we performed functional research to explore the molecular mechanisms underlying such genetic marker for vitiligo.

## Results

### Effects of *AHR* −129C > T polymorphism on *AHR* transcriptional activity

**Figure 1:**
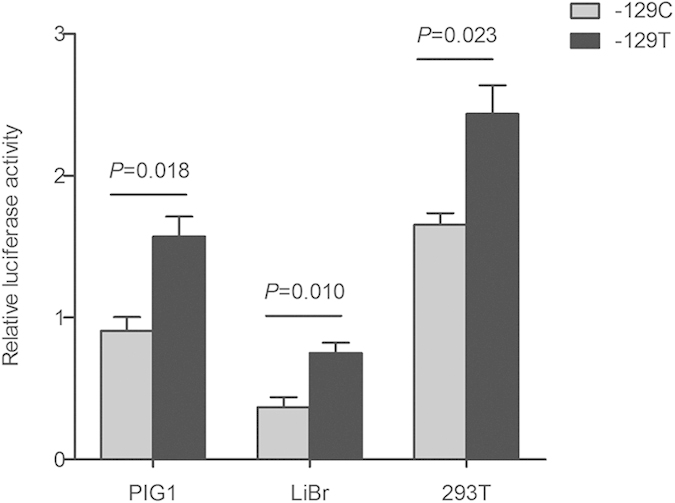
Effects of the −129C > T polymorphism on *AHR* promoter activity. The luciferase reporter plasmids (pGL3-Basic) containing −129C or −129T allele were transiently transfected in PIG1, LiBr and 293T cells. The luciferase activity was normalized against the Renilla luciferase, compared with the construct counterpart. Each column represents Mean ± SEM of three independent experiments.

To assess the *AHR* promoter activity related to −129C > T polymorphism, C or T promoter constructs were transiently transfected in human normal melanocyte PIG1 cells, human malignant melanoma LiBr cells and human embryonic kidney 293T cells. As shown in Fig. 1, the vectors with −129T allele had enhanced relative luciferase activity compared with that of those with −129C allele (*P* = 0.018 for PIG1 cells; *P* = 0.010 for LiBr cells; *P* = 0.023 for 293T cells). These results suggest that the *AHR* −129T allele possesses an increased *AHR* transcriptional activity.

### Impacts of *AHR* −129C > T polymorphism on SP1 binding to *AHR* promoter

**Figure 2:**
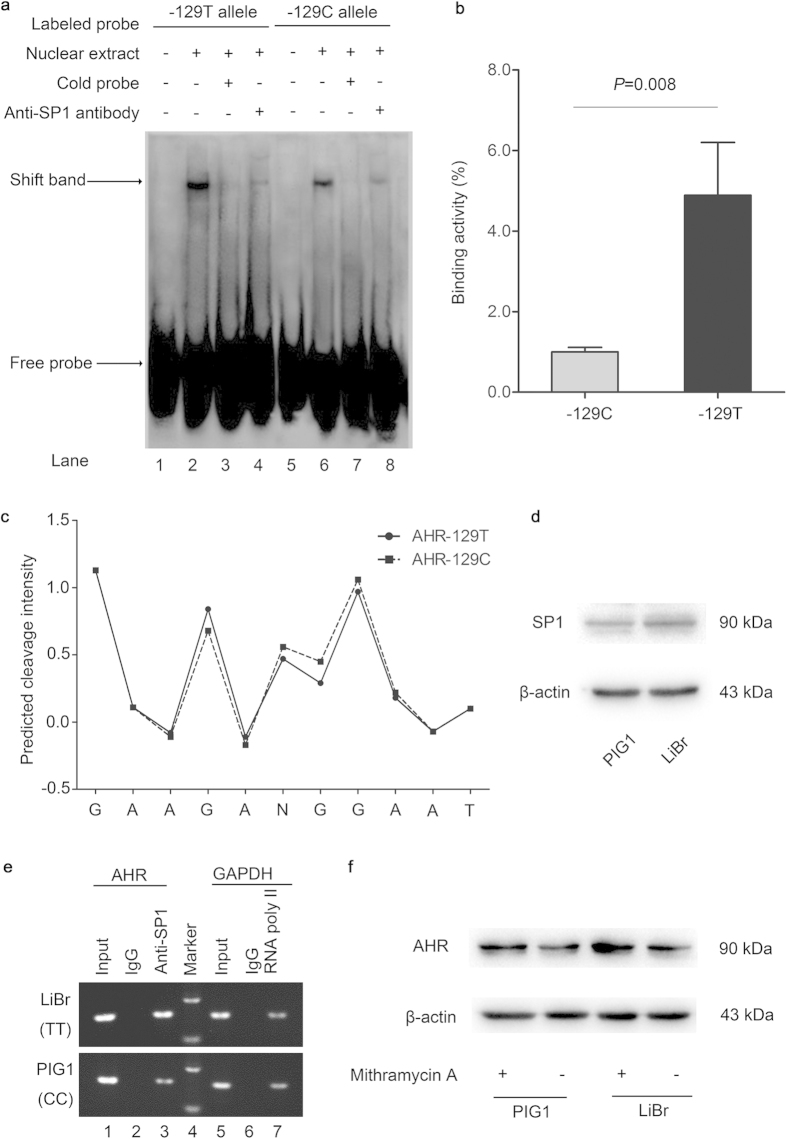
Analysis of SP1 binding to the *AHR* promoter around −129C > T polymorphism. (**a**) Electrophoretic mobility shift assay with biotin-labeled either −129C or −129T probes and PIG1 cell nuclear extracts. Lanes 1 and 5, mobilities of the labeled −129C or −129T probes without nuclear extracts; lanes 2 and 6, mobilities of the labeled −129C or −129T probes with nuclear extracts in the absence of competitor; lanes 3 and 7, shifted bands were abolished by 100-fold unlabeled cold probes; lanes 4 and 8, super shift assays incubating with anti-SP1 antibody showed the supershifted protein complex. (**b**) Quantification of bands in Fig. 2a (lanes 2 and 6) was done using ImageJ software (National Institutes of Health), with the C allele binding being defined as 100%. Results are from three independent experiments with similar results. (**c**) Allelic difference in DNA surface structure was detected at *AHR* −129C > T polymorphism by using predicted hydroxyl radical cleavage patterns. (**d**) Western blots showed that PIG1 and LiBr cells expressed relative levels of SP1 protein. (**e**) Chromatin immunoprecipitation assay with PIG1 cells (−129CC genotype) and LiBr cells (−129TT genotype) in the presence of anti-SP1 (lane 3), anti-RNA Polymerase II (positive control; lane 7) antibodies, or control IgG (negative control; lane 2 and 6). (**f**) The SP1 inhibitor mithramycin A suppressed the expression of AHR protein in PIG1 and LiBr cells, measured by western bolts. These data confirm the biological interaction between SP1 and *AHR* promoter influenced by the −129 C > T polymorphism.

Both nucleotide sequences and DNA shape influence specific binding of proteins to DNA^24^,^25^. The hydroxyl radical cleavage pattern embodies information on sequence-dependent variation in DNA structure, including solvent accessibility, minor groove width and electrostatic potential^26^,^27^. Where the minor groove is wide, and deoxyribose backbone hydrogens are exposed, cleavage intensity is high; where the groove is narrow, and backbone hydrogens are diminished in exposure, cleavage intensity is low^27^,^28^. Minor groove width and electrostatic potential are important for protein binding^25^. Specifically, narrowing of the groove is associated with more negative electrostatic potential, which is beneficial for proteins to insert side chains with positive charge into the groove^28^,^29^. To test the effect of *AHR* −129C > T variant on DNA local structure, bioinformatic analysis was performed using the OH Radical Cleavage Intensity Database (ORChID) and showed different hydroxyl radical cleavage patterns among variants of the *AHR* −129C > T polymorphism. As shown in Fig. 2c, the T allele with relatively lower score reflects narrowing of the DNA backbone, which might function as an electrostatic groove to further improve the DNA-protein interaction.

To detect the role of −129C > T polymorphism in the interplay between *AHR* promoter and transcriptional regulators, we carried out electrophoretic mobility shift assays (EMSA) and found the DNA-protein complex with stronger intensity for the −129T allele than −129C allele (Fig. 2a, lanes 2 and 6; *P* = 0.008, Fig. 2b), which was abolished by excessive unlabeled probes (Fig. 2a, lanes 3 and 7). Since the *AHR* −129C > T polymorphism is close to the binding site of SP1, we then conducted supershift assays and detected that anti-SP1 antibody supershifted the C and T allele-specific bands (Fig. 2a, lanes 4 and 8), implying that this promoter variant could modify allele-specific binding affinity of SP1. We next performed chromatin immunoprecipitation assays (ChIP) to verify these observations within PIG1 cells (−129CC) and LiBr cells (−129TT). Both cell lines expressed comparable levels of SP1 protein (Fig. 2d). Similarly, −129TT genotype had higher abundance in the interaction between SP1 and *AHR* promoter than that of −129CC genotype (Fig. 2e, lane 3). To affirm the role of SP1 activation in AHR expression, PIG1 and LiBr cells were treated with 100 nM mithramycin A, a specific SP1 inhibitor that competitively binds to the SP1-binding sites^30^. The basal expression of AHR protein was relatively higher in LiBr cells (−129TT) than PIG1 cells (−129CC), and mithramycin A treatment suppressed AHR protein expression in PIG1 and LiBr cells (Fig. 2f). Taken together, these data reveal that SP1 prefers to bind to the *AHR* promoter containing −129T allele rather than −129C allele.

### *AHR* and *SP1* transcript levels in vitiligo patients and controls with different genotypes for the *AHR* −129C > T polymorphism

**Figure 3:**
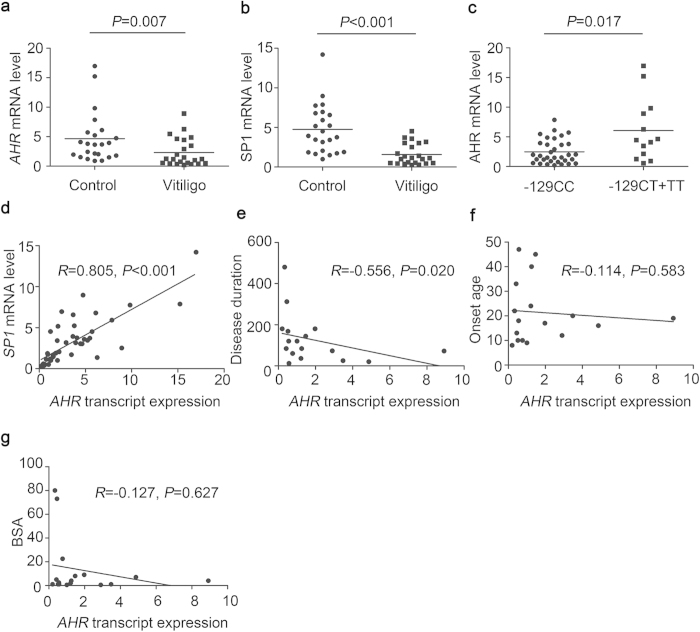
Differential mRNA levels of *AHR* and *SP1* in individuals carrying different *AHR* −129 genotypes. (**a**) Transcript level of *AHR* was significantly declined in vitiligo patients compared with controls. (**b**) Significant difference in *SP1* mRNA level between vitiligo patients and controls was detected. (**c**) Individuals with −129CT/TT genotypes expressed higher level of *AHR* mRNA than those in the −129CC group. (**d**) *AHR* transcript expression was positively correlated with *SP1* mRNA level. Spearman’s correlation analysis of *AHR* mRNA expression to disease duration (month; **e**), onset age (year; **f**) and body surface area involvement (BSA, %; **g**) was conducted in 17 vitiligo patients with integrity data of clinical characteristics (n = 13 for −129CC and n = 4 for −129CT + TT in vitiligo patients).

To investigate the impact of −129C > T polymorphism on *AHR* transcript level and the correlation between *SP1* and *AHR* expressions, we measured the *AHR* and *SP1* mRNA levels in the peripheral blood mononuclear cells (PBMCs) from 23 vitiligo patients and 23 age- and sex-matched controls harboring different *AHR* −129 genotypes (n = 16 for CC, n = 6 for CT and n = 1 for TT in vitiligo patients; n = 17 for CC, n = 5 for CT and n = 1 for TT in controls; Hardy–Weinberg equilibrium test: χ^2^ = 0.571, *P* = 0.450). We observed positive correlation between *AHR* and *SP1* expressions (*R* = 0.805, *P* < 0.001, Fig. 3d), both of which were obviously diminished in vitiligo patients compared with controls (*P* = 0.007 for *AHR*, Fig. 3a; *P* < 0.001 for *SP1*, Fig. 3b). Moreover, the *AHR* transcript expression was negatively correlated with total disease duration (*R* = −0.556, *P* = 0.020, Fig. 3e), but no significant relevance with disease onset age and body surface area (BSA) involvement (Fig. 3f,g). As expected, individuals with protective −129TT/CT genotypes had higher *AHR* level than those with −129CC genotype (6.059 ± 1.467 vs. 2.476 ± 0.356, *P* = 0.017, Fig. 3c). These findings implicate that the *AHR* −129C > T polymorphism reflects on the level of *AHR* transcript expression.

### Association between the *AHR* −129C > T polymorphism and serum cytokine levels in vitiligo

**Figure 4:**
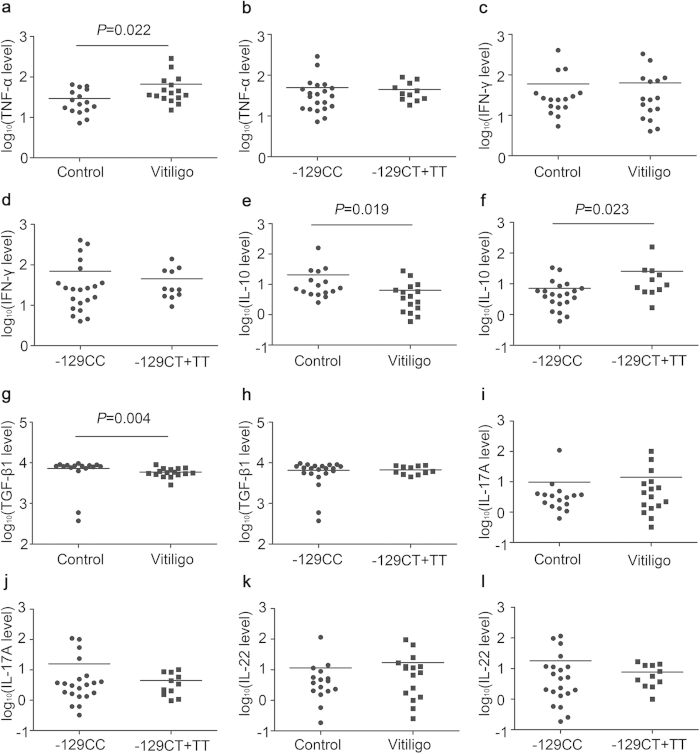
Impacts of the *AHR* −129C > T polymorphism on serum cytokine levels in vitiligo. The amounts of TNF-α (**a**), IFN-γ (**c**), IL-10 (**e**), TGF-β1 (**g**), IL-17A (**i**) and IL-22 (**k**) in the serum from vitiligo patients and controls were determined by ELISA. To compare cytokine expressions between −129CC risk genotype group and −129CT + TT protective genotype group, genetic analysis data were acquired from cases and controls together (**b**,**d**,**f**,**h**,**j** and **l**). The y axis is in log10 scale.

Because T cell-mediated autoimmunity and altered cytokines are involved in vitiligo as well as AHR plays a pivotal role in T cell development and cytokine production, mutation or abnormal expression of AHR may influence cytokine levels associated with vitiligo. We therefore examined the major cytokines of T helper and regulatory T cells in 16 vitiligo patients and 16 age- and sex-matched controls carrying various *AHR* −129 genotypes (n = 10 for CC, n = 4 for CT and n = 2 for TT in vitiligo patients; n = 11 for CC, n = 4 for CT and n = 1 for TT in controls; Hardy–Weinberg equilibrium test: χ^2^ = 0.515, *P* = 0.473). We detected significantly increased TNF-α concentration and decreased levels of IL-10 and TGF-β1 in the serum of vitiligo patients compared with controls (29.451 ± 4.748 ng/ml vs. 66.500 ± 17.805 ng/ml, *P* = 0.022 for TNF-α; 20.649 ± 9.514 vs. 6.375 ± 1.885 ng/ml, *P* = 0.019 for IL-10; 7258.105 ± 687.912 ng/ml vs. 5895.036 ± 365.716 ng/ml, *P* = 0.004 for TGF-β1; Fig. 4a,e,g). No difference was observed between the cases and controls in the levels of IFN-γ, IL-17A and IL-22 (Fig. 4c,i,k). Furthermore, individuals with −129TT/CT genotypes had greatly enhanced IL-10 expression compared with the group with −129CC genotype (7.118 ± 1.869 ng/ml vs. 25.719 ± 13.565 ng/ml, *P* = 0.023; Fig. 4f). However, the *AHR* −129C > T polymorphism seems not to be related to other cytokines (Fig. 4b,d,h,j,l). The evidence supports that the *AHR* −129C > T polymorphism might be associated with IL-10 production.

## Discussion

In the current study, we investigated the molecular mechanisms of *AHR* functional variation (−129C > T polymorphism) underlying its association with vitiligo. Our data demonstrated that the protective T allele of this polymorphism dramatically enhanced *AHR* promoter activity through promoting the binding activity of SP1. We also found decreased *AHR* transcript expression in the PBMCs of vitiligo patients, which was negatively correlated with disease duration. Further genetic analysis showed that −129T carriers had higher *AHR* expression than −129C carriers. We further detected AHR related cytokines in vitiligo patients and observed declined serum IL-10 level and its association with the *AHR* −129C > T variant. Thus, our scientific evidence strengthens our previous finding^17^ and further clarifies that the *AHR* −129C > T polymorphism is indeed a functional genetic marker for vitiligo.

In this report, we first proved the contribution of −129C > T variation to *AHR* promoter function, which might influence *AHR* transcription by altering its interaction with transcriptional regulators. Human *AHR* gene contains features of a housekeeping gene as possessing GC-rich regions^31^. The core promoter region (−1 ∼ −250) has a GC content of 70%, containing several putative binding sites for SP1^32^. Deletion of the SP1 recognition site reduced basal promoter activity of human *AHR* gene to 20%^21^. In addition, impaired interaction between SP1 and *AHR* promoter caused by SP1 inhibitor or *AHR* hypermethylation led to AHR downregulation^22^,^23^,^33^. Consistent with previous study, we also found that SP1 inhibitor down-regulated AHR expression in PIG1 and LiBr cells. These results suggest that the SP1-binding motif is sufficient for maximal constitutive expression of *AHR* genes. Given the pivotal role of SP1 in *AHR* expression and *AHR* −129C > T variation located nearby the core binding site for SP1, we then performed EMSA and ChIP assays and observed the −129T allele with higher SP1 binding affinity than the −129C allele. Li D. *et al.* previously reported that the −129C > T variant interfered the binding activity of nuclear factor 1-C, which might down-regulate *AHR* transcription *via* antagonizing SP1 occupancy^34^. Although the mutual effect of SP1 and other regulators on *AHR* transcription remains unclear, it is conceivable that the *AHR* −129C > T variant may affect the binding of SP1 to the *AHR* promoter in a direct or indirect manner.

The association between *AHR* expression and vitiligo remains to be elucidated. Gene expression analysis of PBMCs from independent cases and controls showed apparently decreased *AHR* mRNA level in vitiligo, which was clearly negatively correlated with disease duration of vitiligo patients, suggesting that down-regulation of *AHR* transcription is relative to vitiligo. We also found that declined *SP1* mRNA level in vitiligo patients and a strongly positive correlation between *SP1* and *AHR* expressions. It has been reported that the expression levels of SP1 and AHR vary greatly among different tissues^21^, but highest mRNA levels of *SP1* and *AHR* were observed in the same tissues, such as thymus, lung, and spleen^35^,^36^. Differential SP1 expression and abnormal SP1 binding activity were demonstrated to mediate reduced expressions of AHR and its target genes in human breast cancer cells^22^,^37^. In order to investigate whether the regulatory role of −129C > T mutation in SP1 binding affinity and *AHR* promoter activity could eventually influence *AHR* transcript expression, we performed genetic analysis showing that the protective −129T allele is associated with elevated expression of *AHR* mRNA. Both *in-vitro* and *in-vivo* transcriptional analyses provided consistent results, indicating the potential effect of −129C > T mutation on *AHR* expression, which might in turn influence its function in regulating melanogenic factors including TYR, TYRP, SCF and C-Kit^14^,^15^. Preliminary studies have implicated that abnormalities of TYR and TYRP, such as genetic mutation, decreased expression, altered folding or maturation and aberrant retention, may lead to increased toxic metabolites and subsequent melanocyte apoptosis in vitiligo^38^,^39^. TYRP could prevent premature melanocyte death in animals^39^. Moreover, Mutation of SCF or C-Kit caused hair hypopigmentation in mice^40^, and aberrant SCF/C-Kit pathway contributed to vitiligo and piebaldism in human^41^,^42^,^43^,^44^. SCF could protect primary human melanocytes against apoptosis and promote repigmentation in vitiligo mouse model^45^,^46^. Accordingly, it could be speculate that the *AHR* −129C > T polymorphism might be associated with vitiligo through influencing *AHR* expression and its downstream melanogenic factors.

To further understand another significance of *AHR* −129C > T polymorphism, we assessed the impact of this functional variant on the immune cytokines in vitiligo. Consistent with previous findings^47^,^48^, we found increased TNF-α concentration and declined levels of IL-10 and TGF-β1 in the serum of vitiligo patients. Further genetic analysis showed that IL-10 was strikingly higher in subjects carrying *AHR* −129T allele than −129C allele. Recent studies have demonstrated that AHR could affect IL-10 expression *via* mediating the differentiation of Treg cells^49^,^50^. AHR activation promotes Treg cells development by diverse mechanisms, including transcription regulation^13^, epigenetic modification^51^, cooperation with transcription factors Smad1 (SMAD family member 1) and Aiolos (IKAROS family zinc finger 3)^50^, inhibition of IL-2^52^ and limitation of STAT1 (signal transducer and activator of transcription 1) activation^53^. These Treg cells induced by AHR pathway were shown to ameliorate autoimmune diseases such as type I diabetes^54^, multiple sclerosis^13^, colitis^51^ and graft versus host disease^55^. Further, naive T cells from *Ahr* null mice inefficiently generated Tregs with decreased IL-10 levels^53^,^56^, indicating a specific role of AHR in Treg generation and IL-10 production. Recently, abnormal Tregs were identified in vitiligo, with evidence of reduced number, impaired skin homing, defective function and declined IL-10 expression^48^,^57^,^58^,^59^. Consequently, our data of genetic analysis imply that the *AHR* −129C > T mutation might be related to vitiligo partially by affecting IL-10 production, which further supports previous findings about AHR function in immune regulation. Nevertheless, immune system contains many biological structures and complex processes, which could be modulated by mutual effort of multiple factors including AHR. Thus, functional studies may be needed to explore the regulatory mechanisms of AHR pathway in the immune pathology of vitiligo further.

There are several limitations within our study. First, owing to absence of melanocytes in vitligo epidermis and Koebner phenomenon exiting in vitiligo, it is hard to acquire primary melanocytes or tissue samples from vitiligo patients and we therefore used PBMCs instead of melanocytes to confirm these findings. Second, multiple genes and various environmental factors make collaborative contribution to vitiligo. These highlight the need to explore the gene-gene or gene-environment interaction in vitiligo in our future genetic studies.

In summary, we provide evidence that *AHR* –129C > T variant could lead to allele-specific binding of SP1 to *AHR* promoter and further modify its transcription and downstream effectors in vitiligo. Therefore, our findings have not only advanced our understanding on AHR physiological function but also provided significant insight into the pathogenesis of degenerative disorders or autoimmune diseases including vitiligo.

## Methods

### *In silico AHR* promoter analysis

ORChID (dna.bu.edu/orchid) was used to predict the hydroxyl radical cleavage pattern of local variation in solvent-accessible surface area of duplex DNA, and provided information on the local structural DNA profiles from nucleotides sequence containing the *AHR* −129C > T polymorphism.

### Cell culture

Human normal melanocyte PIG1 cell line (a gift from Dr Caroline Le Poole, Loyola University Chicago, Maywood, IL, USA) was cultured in Medium 254 (Cascade Biologics/Invitrogen, Portland, OR, USA) supplemented with Human Melanocyte Growth Supplement (Cascade Biologics/Invitrogen, Portland, OR, USA) and 5% Fetal Bovine Serum (FBS; Invitrogen, Carlsbad, CA, USA). Human embryonic kidney 293T cell line and human malignant melanoma LiBr cell line were maintained in Dulbecco’s Modified Eagle Medium: Nutrient Mixture F-12 (Invitrogen, Carlsbad, CA, USA) supplemented with 10% FBS. For SP1 inhibition, PIG1 and LiBr cells were treated with 100 nM chemical inhibitor mithramycin A (Enzo Life Sciences, Farmingdale, NY) or control vehicle DMSO (Sigma-Aldrich Corp., St. Louis, MO) for 48 h.

### Blood samples

For assays of gene expression, 23 patients with vitiligo and 23 sex- and age-matched control subjects were enrolled in the present study. We obtained 10 ml of peripheral blood from each participant for PBMCs isolation as described^58^. For cytokines measurement, we used 4 ml of peripheral blood from 16 vitiligo patients and 16 sex- and age-matched health subjects for serum collection. The detailed methods of recruiting subjects and genotyping of *AHR* −129C > T variant have been described previously^17^. In particular, information on demographics and other characteristics, including disease duration, onset age and BSA, was obtained using questionnaires (details shown in Supplementary Table S1 online). All the subjects gave written informed consent. The study was approved by the ethics review board of the Fourth Military Medical University and was conducted according to the Declaration of Helsinki Principles.

### Construction of reporter plasmids

A 534-bp DNA fragment (−534 to −1) of human *AHR* promoter region was directly synthesized with added KpnI and MluI sites at 5′ ends in forward and reverse sequences, respectively (Cnservice Invitrogen, Shanghai, China). The DNA sequence was inserted between KpnI and MluI sites in the firefly luciferase reporter vector pGL3-Basic (Promega, Madison, WI, USA). The −129C > T polymorphism was introduced into the recombinant pGL3 reporter vector by performing site-directed mutagenesis. The sequence of constructs was confirmed by direct DNA sequencing.

### Transient transfections and dual-luciferase assay

For transfections, PIG1, LiBr and 293T cells were plated onto 24-well plates, respectively. The cells were transfected with 0.8 μg of the recombinant pGL3 reporter vector with either −129C or −129T allele using Lipofectamine 2000 Transfection Reagent (Invitrogen, Carlsbad, CA, USA). The pGL3-Basic vector without an insert and pGL3-Control vector (Promega, Madison, WI, USA) were used as a negative control and a positive control, respectively. All plasmids were cotransfected with 0.02 μg of pRL-SV40 vector (Promega, Madison, WI, USA) as an internal standard. At 48 h after transfection, cells were collected and luciferase activity was measured with Dual-Luciferase Reporter Assay System (Promega, Madison, WI, USA) according to the manufacturer’s protocols. Luciferase activity was normalized against the activity of pRL-SV40 with the Renilla luciferase gene. Independent triplicate experiments were done for each plasmid construct.

### EMSA assay

The DNA-protein interactions were detected using LightShift Chemiluminescent EMSA Kit (Thermo Fisher Scientific, Waltham, Massachusetts, USA). The biotin labeled and unlabeled double-stranded oligonucleotides corresponding to a 30-bp sequence 5′-BIO-AAGAC/TGGAATGGAATCCAGATGGGCGGGGG-3′ (SP1 binding site underlined) were synthesized by Viagene Biotech Inc (Viagene Biotech Inc, Tampa, Florida, USA). Nuclear extracts (10 μg) were isolated from PIG1 cells using Viagene Subcellular Protein Fraction Kit (Viagene Biotech Inc, Tampa, Florida, USA) and protein concentration was determined by BCA Protein Assay Kit (Thermo Fisher Scientific, Waltham, Massachusetts, USA). For each gel shift reaction (15 μl), a total of 300 fmol labeled probes were combined with 10 μg of nuclear extract, 1.5 μg of poly (deoxyinosinic-deoxycytidylic acid) and 10 × binding buffer. For competition assays, a 100-fold molar excess of unlabeled either −129C or −129T probes was preincubated for 20 min at room temperature with nuclear extracts before addition of the labeled probes. For each supershift reaction (15 μl), 3.5 μl of anti-SP1 antibody (Abcam Inc., Cambridge, MA, USA) was incubated with nuclear extracts at 4 °C for 15 min, followed by an additional incubation for 20 min at room temperature with the labeled probes. After incubation, samples were separated on a native non-denaturing 6.5% polyacrylamide gel and then transferred to a nylon membrane. The positions of biotin-labeled probes in the membrane were detected by a chemiluminescent reaction with the stabilized streptavidin– horseradish peroxidase conjugate according to the manufacturer’s instructions and visualized by autoradiography.

### ChIP assay

Before ChIP assay, PIG1 cells and LiBr cells were genotyped and sequenced to be CC and TT homozygous, respectively, which were performed similarly to our previous procedures (see Supplementary Fig. S1 online)^17^. ChIP assays were performed with ChIP-IT Express Kit (Active Motif, Carlsbad, USA) according to the manufacturer’s protocols. Briefly, 1 × 10^6^ of PIG1 cells and LiBr cells were treated with 2.7% formaldehyde for 10 min, followed by 2.5 M glycine stop solution. Cells were then collected and centrifuged at 4 °C and resuspended in lysis buffer for 30 min. The extracts were sonicated, and the DNA was sheared using enzymatic shearing cocktail until the DNA fragments were 200 ∼ 500-bp in size. Sheared chromatin (10 μl) was incubated with protein G magnetic beads and 3 μg of rabbit polyclonal anti-SP1 antibody (Abcam Inc., Cambridge, MA, USA), rabbit control IgG (Santa Cruz Biotechnology) or rabbit polyclonal anti-RNA polymerase II antibody (Abcam Inc., Cambridge, MA, USA) overnight at 4 °C. After extensive washing of the beads, the samples were reverse cross-linked and treated with ribonuclease A and proteinase K. After DNA purification, the immunoprecipitated chromatin fraction was amplified by PCR with primers specific for the *AHR* promoter (see Supplementary Table S2 online) and control primers for the *GAPDH* promoter. The PCR products were analyzed on a 2.0% agarose gel.

### Real-time quantitative RT-PCR

The total RNA was extracted from the PBMCs of vitiligo patients and controls with Trizol Reagent (Invitrogen, Inc.) and then converted to cDNA using PrimeScript^TM^ RT Master Mix (TaKaRa, Inc.). The mRNA levels of *AHR*, *SP1* and *ACTB* were measured by real-time quantitative PCR using specific primers (see Supplementary Table S2 online). Fold changes were normalized by the level of *ACTB* expression, and each assay was done in triplicate.

### Western blotting

SP1 and AHR protein expressions in PIG1 and LiBr cells were examined by Western blots analysis. Western blots (50 μg protein/lane) were performed as described previously^30^, and probed with monoclonal antibodies specific for AHR or SP1 proteins (Abcam Inc., Cambridge, MA, USA). Hybridization signals for AHR and SP1 were normalized to hybridization signal for β-actin (Abcam Inc., Cambridge, MA, USA).

### Measurement of cytokine concentrations

The amounts of TNF-α, IFN-γ, IL-10, TGF-β1, IL-17A and IL-22 in the serum from each participant were determined using a corresponding ELISA kit (R&D systems, Bender MedSystems, Minneapolis, MN, USA) according to the manufacturer’s instructions.

### Statistical analysis

Student’s *t* test was performed to evaluate the difference in luciferase activities between different constructs. Unpaired *t* test with Welch’s correction was used to assess the different DNA-protein binding affinities between −129T and −129C probes. Non-parametric Mann–Whitney U-test was applied to analyze the results of peripheral *AHR* and *SP1* mRNA expressions and serum cytokine concentrations between vitiligo patients and controls with different *AHR* −129 genotypes. Correlation studies were performed using Pearson’s correlation test. All tests were two-sided by using SPSS software (Version 13.0, SPSS Inc., Chicago, USA). Statistical significance was defined as *P* < 0.05.

## Additional Information

**How to cite this article**: Wang, X. *et al. AHR* promoter variant modulates its transcription and downstream effectors by allele-specific *AHR*-SP1 interaction functioning as a genetic marker for vitiligo. *Sci. Rep.* **5**, 13542; doi: 10.1038/srep13542 (2015).

## Supplementary Material

Supplementary Information
