# Supplementary material for: AHR promoter variant modulates its transcription and downstream effectors by allele-specific AHR-SP1 interaction functioning as a genetic marker for vitiligo
Source: Sci Rep. 2015 Sep 15;5:13542. doi: 10.1038/srep13542 (PMC4570213; doi:10.1038/srep13542)
Supplement: Supplementary Information [file srep13542-s1.pdf]

Supplementary information

AHR promoter variant modulates its transcription and downstream effectors by allele-specific *AHR*-SP1 interaction functioning as a genetic marker for vitiligo

Xiaowen Wang[1,2], Kai Li[1,2], Ling Liu[1,2], Qiong Shi[1], Pu Song[1], Zhe Jian[1], Sen Guo[1], Gang Wang[1], Chunying Li[1*] & Tianwen Gao[1*]

[1]Department of Dermatology, Xijing Hospital, Fourth Military Medical University, Xi'an, Shaanxi, China

[2]Xiaowen Wang, Kai Li and Ling Liu contributed equally to this work.

Supplementary Fig. S1 Genotypes and sequence analysis of *AHR* -129C>T variation in PIG1 and LiBr cells. (a) Genotyping the -129C>T polymorphism in normal human melanocyte cell line (PIG1) and various human melanoma cell lines (A2085, P70 1676, LiBr, A378, 67 and 140). (b) Sequencing the *AHR* polymerase chain reaction products of PIG1 and LiBr cells.

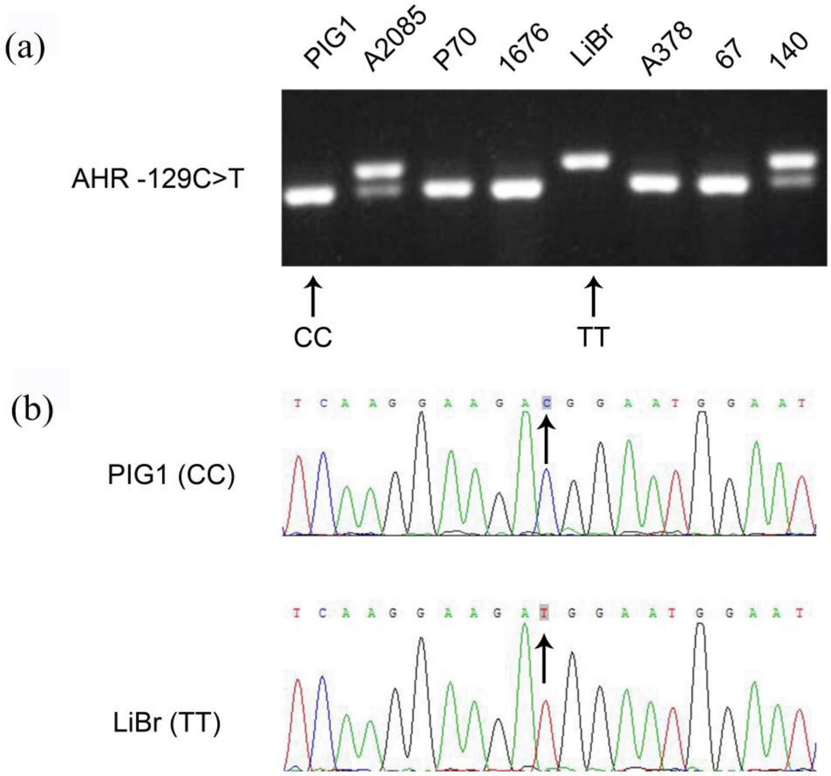

Supplementary Table S1. Primary information for disease duration, onset age and body surface area of vitiligo patients.

Patient ID	Disease duration (month)	Onset age (year)	Body surface area (%)
1	180	8	1.0
2	480	22	80.0
3	84	33	5.0
4	312	13	73.0
5	168	10	1.0
6	120	18	2.5
7	12	47	1.0
8	60	10	22.5
9	120	9	0.5
10	36	24	2.0
11	84	40	4.0
12	144	45	8.0
13	180	17	9.0
14	73	12	0.5
15	25	20	1.0
16	20	16	7.0
17	72	19	4.0

Supplementary Table S2. Primer sequences used for ChIP assay and real-time quantitative PCR amplification.

Assay	Gene	Primer sequences (5’→3’)		Product size (bp)
		Forward primer	Reverse primer	
ChIP	*AHR*	CCCTCAAGGAAGACGGAATGG	CCGGCTGAATAGCAGGAGCA	122
RT-PCR	*AHR*	ACATCACCTACGCCAGTCGC	TCTATGCCGCTTGGAAGGAT	101
	SP1	ATGATGACACAGCAGGTGGA	TTGCCATACACTTTCCCACA	190
	ACTB	TCATGAAGTGTGACGTGGACATC	CAGGAGGAGCAATGATCTTGATCT	156
